# Promotion of Specific Single-Transverse-Mode Beam Characteristics for GaSb-Based Narrow Ridge Waveguide Lasers via Customized Parameter Design

**DOI:** 10.1186/s11671-022-03758-5

**Published:** 2022-12-07

**Authors:** Tianfang Wang, Chengao Yang, Yihang Chen, Jianmei Shi, Hongguang Yu, Xiangbin Su, Yu Zhang, Yingqiang Xu, Zhichuan Niu

**Affiliations:** 1 State Key Laboratory for Superlattices and Microstructures, Institute of Semiconductors, Chinese Academy of Sciences, Beijing, China; 2 Center of Materials Science and Optoelectronics Engineering, University of Chinese Academy of Sciences, Beijing, China

**Keywords:** GaSb-based laser, Single transverse mode, Optimized ridge waveguide

## Abstract

GaSb-based single-transverse-mode narrow ridge waveguide (RW) lasers with high power and simultaneous good beam quality have broad application prospects in the mid-infrared wavelength region. Yet its design and formation have not been investigated systematically, while the beam characteristics that affect their suitability for specific applications remain rarely analyzed and optimized. The present work addresses these issues by theoretically establishing a waveguide parameter domain that generalizes the overall possible combinations of ridge widths and etch depths that support single-transverse-mode operation for GaSb-based RW lasers. These results are applied to develop two distinct and representative waveguide designs derived from two proposed major optimization routes of model gain expansion and index-guiding enhancement. The designs were evaluated experimentally based on prototype 1-mm cavity-length RW lasers in the 1950 nm wavelength range, which were fabricated with waveguides having perpendicular ridge and smooth side-walls realized through optimized dry etching conditions. The model gain expanded RW laser design with a relatively shallow-etched (i.e., 1.55 $\upmu$m) and wide ridge (i.e., 7 $\upmu$m) yielded the highest single-transverse-mode power to date of 258 mW with a narrow lateral divergence angle of 11.1$^\circ$ full width at half maximum at 800 mA under room-temperature continuous-wave operation, which offers promising prospects in pumping and coupling applications. Meanwhile, the index-guiding enhanced RW laser design with a relatively deeply etched (i.e., 2.05 $\upmu$m) and narrow ridge (i.e., 4 $\upmu$m) provided a highly stable and nearly astigmatism-free fundamental mode emission with an excellent beam quality of M$^2$ factor around 1.5 over the entire operating current range, which is preferable for seeding external cavity applications and complex optical systems.

## Introduction

Semiconductor diode lasers operating in the mid-infrared (mid-IR) 2–4 $\upmu$m wavelength region are of considerable interest owing to their excellent inherent properties of small footprint and low cost, and broad application prospects in a variety of fields, such as laser spectroscopy, free-space communication, material processing, and medical therapy [1–3]. Among these mid-IR diode lasers, GaSb alloys are the most promising material systems [3–5]. In fact, GaSb-based broad area (BA) lasers operating in the 2 $\upmu$m wavelength region have been reported with a maximum output power near 2 W [1, 6, 7]. Nonetheless, relatively few studies have focused on the development of narrow RW GaSb-based lasers with single-transverse-mode emission.

The potential for higher output power with simultaneously good beam quality makes GaSb-based single-transverse-mode narrow ridge waveguide (RW) lasers ideally suited light sources for various scientific and commercial applications, such as pumping rare-earth-doped fiber amplifiers and solid-state lasers [8–10], seeding external cavity lasers [11, 12], and nonlinear frequency conversion [13]. In addition, narrow RW structure is also widely employed in various laser devices, such as distributed-feedback (DFB) lasers [3, 14], distributed Bragg reflector (DBR) lasers [15, 16], superluminescent diodes (SLD) [5, 17], semiconductor optical amplifiers (SOA) [18], and tapered lasers [19], owing to its ability to guarantee high transverse mode purity and stability, which enables the use of simple and low-cost optics for the focusing and coupling of these devices for further utilization.

In fact, various applications emphasize different aspects of the single-transverse-mode beam characteristics. For example, applications that perform beam transformations in complicated optical systems strictly require excellent beam quality, low astigmatism, and highly stable single-transverse-mode emission [9, 12]. In contrast, pumping or coupling applications put higher emphasis on the promotion of optical output power and the compression of beam divergence [10, 18, 19]. Meanwhile, GaSb-based DFB, DBR, or SLD devices require relatively deep ridge etching to obtain a more substantial coupling effect of the grating or curved structure with the active layer [3, 15, 16]. However, these distinctive and even competing demands were often neglected in previous research using GaSb-based single-transverse-mode laser diodes, in which a 5 $\upmu$-m width RW was usually adopted without systematic investigation [2, 4, 5, 17]. Accordingly, a deeper understanding of the ridge waveguide design and formation, as well as their impact on single-transverse-mode beam characteristics, is necessary.

The present work addresses these issues by establishing a waveguide parameter domain that generalizes the overall theoretical possible combinations of ridge widths and etch depths that support single-transverse-mode operation, based on the higher-order mode cutoff condition according to the analysis of the effective-index slab waveguide model that extracted from a 1950-nm GaSb-based narrow RW laser diode. The two dimensions of the waveguide structure design, including transverse mode confinement and guiding mechanism, have been demonstrated. These results are applied to develop two distinct waveguide designs A and B, representing the two major waveguide optimization routes of model gain expansion and index-guiding enhancement, adopting either shallow or deep etching (i.e., 1.55 $\upmu$m or 2.05 $\upmu$m) in conjunction with either a wide or narrow ridge (i.e., 7 $\upmu$m or 4 $\upmu$m), respectively. The impact of the RW designs was evaluated experimentally based on 1-mm-long prototype F-P lasers fabricated with ridge waveguides having steep and smooth side-walls realized through inductively coupled plasma (ICP) dry etching with an optimized Cl$_2$/BCl$_3$/Ar gas proportion of 5:3:1 and radio frequency (RF) power of 50 W. The perpendicular ridge waveguide enables high consistency with the designed waveguide model while benefiting the single-transverse-mode stabilization due to its effective inhibition of the lateral injection carrier leakage and current spreading.

As a result, both laser diode designs A and B exhibited single-transverse-mode operation with high optical power exceeding 150 mW with simultaneous good beam quality of M$^2$ factor lower than 2. The deeply etched, narrow ridge laser (Design A) that includes a robust index-guiding mechanism and tight mode confinement provided highly stable single-transverse-mode emission with ultra-low astigmatism. In contrast, the shallow-etched, wide ridge laser (Design B) with expanded gain profile and mode distribution exhibited the highest ever reported single-transverse-mode output power of 258 mW at 800 mA and 20$^\circ$C under continuous-wave (CW) operation, together with a narrow lateral beam divergence angle of 11.1$^\circ$ full width at half maximum (FWHM). The prominent improvements in terms of different critical optical characteristics obtained via targeting waveguide structural modifications have provided solid evidence for the validity and practicability of the design concepts of this work while effectively promoting the accommodation of GaSb-based single-transverse-mode RW lasers for specific application demands.

## Ridge Waveguide Structure Design, Fabrication, and Optimization

### Laser Structure Epitaxy and Ridge Waveguide Fabrication

**Fig. 1:**
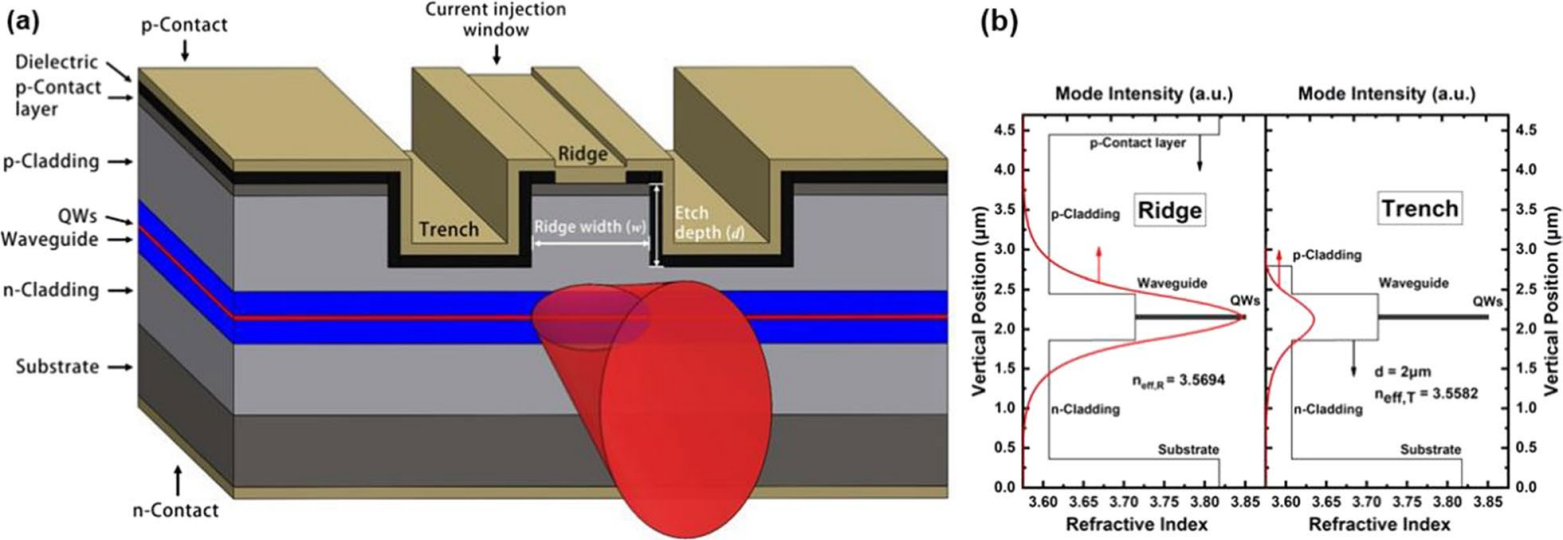
**a** Schematic illustration of the GaSb-based epitaxial layer and narrow RW structures under investigation. **b** Vertical refractive index profiles of the epitaxial structure in the ridge and trench regions with simulated near-field distributions and calculated effective indices of the fundamental mode

The GaSb-based laser epitaxial structure presented in Fig. 1a was grown by solid source molecular beam epitaxy (MBE) on a (100) n-type GaSb substrate. Two compressively strained 13 nm In$_{0.18}$GaSb quantum wells (QWs) separated by a 20-nm Al$_{0.25}$GaAs$_{0.02}$Sb barrier were placed symmetrically between Al$_{0.25}$GaAs$_{0.02}$Sb waveguide layers of a combined thickness of 540 nm. The compositions and the thicknesses of the QWs were optimized for an emission wavelength in the 1.9–2.0 $\upmu$m region. The total thickness of the active region, including the QWs, barrier, and waveguide layers, was about 580 nm. The undoped active region was sandwiched between n-doped and p-doped Al$_{0.5}$GaAs$_{0.04}$Sb cladding layers with thicknesses of 1.5 and 2.0 $\upmu$m, respectively. Finally, a 250-nm highly p-doped GaSb contact layer was grown on top of the p-doped cladding.

The epi-wafer was then fabricated into laser diodes with a narrow RW structure, as shown in Fig. 1a. The ridge waveguide was defined by contact lithography with a pair of 25 $\upmu$-m-wide trenches of depth *d* formed by ICP dry etching into the p-cladding layer on both sides of a narrow ridge of width *w*. A 250-nm SiO$_2$ dielectric layer was then applied to the surface of the etched wafer via plasma-enhanced chemical vapor deposition (PECVD). A current injection window with a width of 2 $\upmu$m narrower than *w* was opened at the center of the ridge through negative photoresist contact lithography and ICP etching. Contact electrode layers composed of Ti/Pt/Au and AuGe/Ni/Au were, respectively, sputtered on the p-type and n-type sides after the wafer was thinned down to 125 $\upmu$m. Thermal annealing was applied to form low-resistance Ohmic contact electrodes. Finally, the RW laser diodes were cleaved, coated with 2$\%$ anti-reflection (AR) and 95$\%$ high reflection (HR) films on the front and rear facets, respectively, and mounted p-side down onto Cu mounts.

#### Waveguide Parameter Design

The effective index method and the slab waveguide model have been employed for the theoretical transverse mode calculation with the epitaxial layer composition and ridge waveguide geometry illustrated in Fig. 1a [20, 21]. In the vertical direction, the epitaxial refractive index profiles of the ridge and trench regions are presented in Fig. 1b with the corresponding simulated fundamental mode near-field distribution and effective index. As can be seen, the 580-nm-thick active region well guaranteed fundamental mode operation with a calculated optical confinement factor (OCF) exceeding 68$\%$, which is far greater than that of the second-order mode of 2.9$\%$. The lateral slab waveguide model was established according to the calculated effective indices of the fundamental mode in the ridge and trench regions, which are denoted herein as $n$ $_{\textrm{eff},R}$ and n$_{\textrm{eff},T}$, respectively. According to Maxwell’s function, the lateral transverse mode eigenvalue equation can be formed with the wavelength $\lambda$ and the waveguide parameters of ridge width *w* and effective index $n$ $_{\textrm{eff}}$. The etching depth *d* is implicated in the $n$ $_{\textrm{eff},T}$, or the effective index step $\Delta$ $n$ $_{\textrm{eff}}$ = $n$ $_{\textrm{eff},T}$ − $n$ $_{\textrm{eff},R}$ as well. For the symmetric etched ridge waveguide structure, an eigenvalue of the fundamental transverse mode always exists for arbitrary waveguide parameters. Meanwhile, the cutoff condition of the second lateral mode eigenvalue yields inequality relationships associated with the ridge width and effective index (etch depth) that guarantee higher-order mode discrimination in the waveguide structure, as follows:

1 $$\begin{aligned} {\left\{ \begin{array}{ll} w \le \dfrac{\lambda }{2\sqrt{n_{\textrm{eff},R}^2-n_{\textrm{eff},T}^2}},or \\ n_{\textrm{eff},T}(d) \le \sqrt{n_{\textrm{eff},R}^2-(\lambda /2w)^2} \end{array}\right. } \end{aligned}$$

**Fig. 2:**
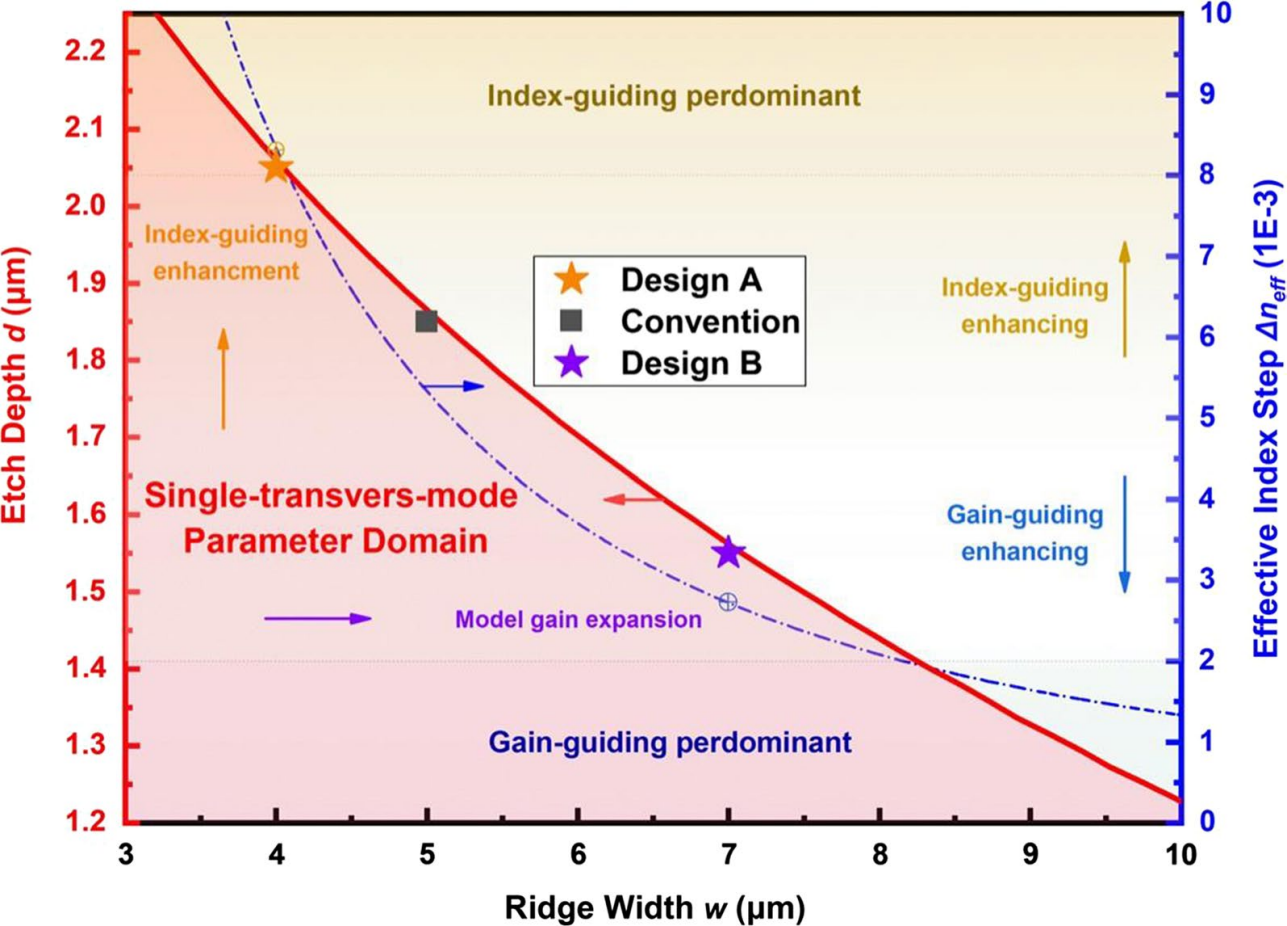
Two-dimensional waveguide parameter domain that supports the single transverse mode operation with Design A (*w* = 4 $\upmu$m, *d* = 2.05 $\upmu$m) and Design B (*w* = 7 $\upmu$m, *d* = 1.55 $\upmu$m), which reflect the two major optimization directions involving index and gain enhancements, respectively. The conventional design (*w* = 5 $\upmu$m, *d* = 1.85 $\upmu$m) is also indicated

The inequality relationships in (1) can be further translated into a waveguide parameter domain presented in Fig. 2, which summarizes the theoretical possibilities of the RW structure parameters that support fundamental mode operation. The figure indicates the two dimensions of the waveguide structure design. The ridge width *w* determines the extent of transverse mode confinement and the pumping region area, while the etch depth *d* (or effective index step $\Delta$ $n$ $_{\textrm{eff}}$) mainly affects the guiding mechanism of the waveguide [22, 23]. An increasing *w* effectively expands the optical mode distribution and the ridge gain profile and therefore benefits the compression of far-field divergence and the promotion of optical power. However, the correspondingly diminished etch depth results in a lower effective index step that leads to the introduction of a stronger gain-guiding mechanism to the waveguide, which exacerbates detrimental effects such as lateral current spreading, thermal lensing, and spatial hole burning that induce transverse mode instabilities [24, 25]. In contrast, the deeply etched ridge waveguide with a corresponding narrow ridge is expected to support robust single-transverse-mode operation and high beam quality due to the predominant index-guiding mechanism. Accordingly, the two optimization routes of the waveguide design, model gain expansion and index-guiding enhancement, have been indicated.

The present work addresses these design concepts by proposing a deep-etched narrower ridge Design A with *w* = 4 $\upmu$m and *d* = 2.05 $\upmu$m, as well as a shallow-etched wider ridge Design B with *w* = 7 $\upmu$m and *d* = 1.55 $\upmu$m, which, respectively, represent the two distinct directions for optimizing the RW structure. The conventional 5 $\upmu$-m-wide RW structure that was widely adopted in the previous research is also presented as a reference for the two major investigated designs. Here, the values of *d* for all the devices were set as large as possible within the fundamental mode operation domain to minimize the impacts of gain-enhancement effects.

**Fig. 3:**
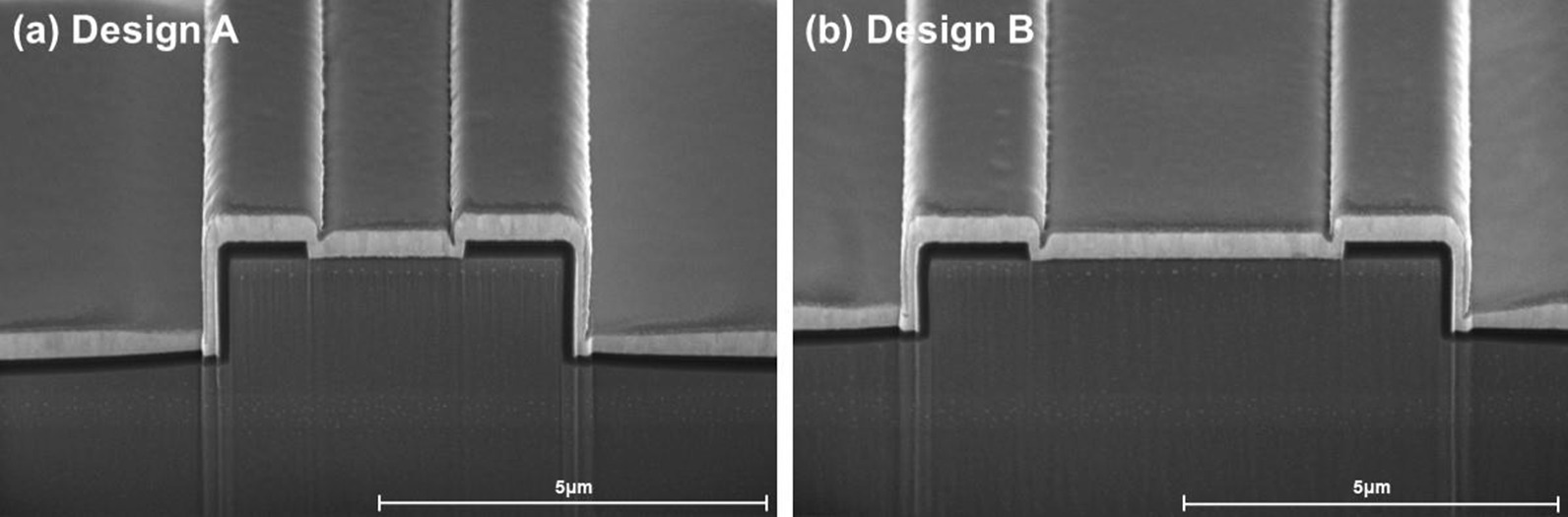
Cross-sectional SEM images of optimized RW lasers: **a** Design A and **b** Design B

#### Ridge Etching Optimization

The ICP dry etching technology plays a significant role in RW formation. The waveguide parameter design is based on the slab waveguide geometry. In order to improve the consistency of the fabricated etching ridge with the designed waveguide parameters and the effective index step model, the ICP dry etching conditions have been optimized. A perpendicular ridge with smooth side-walls has been realized with the Cl$_2$/BCl$_3$/Ar gas proportion of 5:3:1 and an RF power of 50 W, which was confirmed by scanning electron microscopy (SEM) images of the cross-section of the fabricated Designs A and B, as shown in Fig. 3. Designs A and B were fabricated from different pieces of the same epi-wafer. Drastic differences in the ridge width and etch depth of the two waveguide designs can be intuitively recognized in Fig. 3. Measured with the step profiler and SEM method, the fabricated version of Design A exhibited actual values of *w* = 4.04 $\upmu$m and *d* = 2.02 $\upmu$m, while the fabricated version of Design B exhibited *w* = 6.89 $\upmu$m and *d* = 1.47 $\upmu$m.

This optimized ridge etching RW lasers produced a stable single-transverse-mode operation and kink-free light-current characteristics over a working current range of up to 500–800 mA, while RW lasers with conventional trapezium geometries and equivalent waveguide parameters exhibited double-lobed lateral far-field beam patterns at a substantially lower current of 250–400 mA. Since the passive slab waveguide model is known to suffer from detrimental effects in real operating conditions induced by the injection pumping current and heat accumulation, which manifests in the appearance of higher-order lateral modes in lasing emission and kinks in light-current characteristics [21–24]. We attributed the improved single-transverse-mode stability of the perpendicular ridge to the effective inhibition of the injected carrier leakage and lateral current spreading effect. Accordingly, the optimized ridge etching condition supports the effectiveness and practicability of the passive slab waveguide design model in real laser device realization.

## Laser Performances

The performances of RW laser diodes with a 1-mm cavity length and 2$\%$/95$\%$ AR/HR coating on the front and rear facets were measured under continuous-wave (CW) operation at 20 $^\circ$C room temperature unless otherwise specified.

**Fig. 4:**
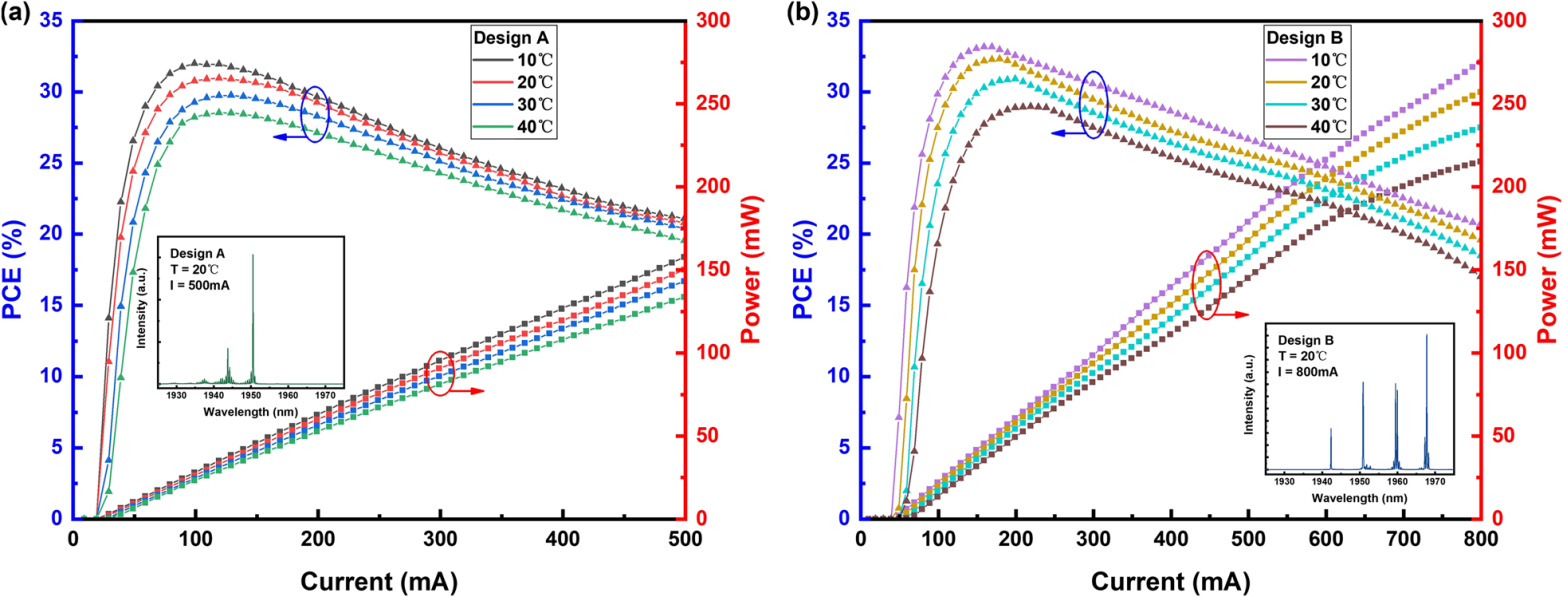
P-I-PCE characteristics of the RW laser designs under CW operation at different heat sink temperatures: **a** Design A and **b** Design B. The insets show the emission spectrums at the maximum operating currents of Designs A and B, respectively

The power–current (P–I) characteristics and power conversion efficiency (PCE) of the fabricated versions of Designs A and B under CW operation and different heat sink temperature are presented in Fig. 4. At room temperature 20 $^\circ$C, the maximum single-transverse-mode output power of Design A was 150 mW at a driving current of 500 mA and that of Design B was the highest ever reported 258 mW at a driving current of 800 mA, limited by catastrophic optical mirror damage (COD). Designs A and B exhibited maximum PCE values of 31.1$\%$ and 32.3$\%$, slope efficiencies of 0.33 and 0.35 W/A, and threshold current densities of 1.40 and 1.45 kA/cm$^{-2}$, respectively. The insets of Fig. 4 present the emission spectrums of Design A and B, the central emission wavelength at an injection current of 500 mA was in the 1950 nm region. Design B realizes remarkable promotion of optical power and efficiency as well as COD occurring current because the wider RW and shallower etching broadened the lateral mode confinement and modal gain profile. However, slight kinks were observed in the P–I curve of Design B. This may have been related to the influence of the gain-enhancing effect obtained at the relatively low effective index step, which made Design B more susceptible to current and temperature perturbations than the predominantly index-enhancing Design A. Meanwhile, Design A obtained a lower threshold current density, which can be attributed to the less pronounced lateral current spreading effect with the thinner residual p-cladding thickness [26].

**Fig. 5:**
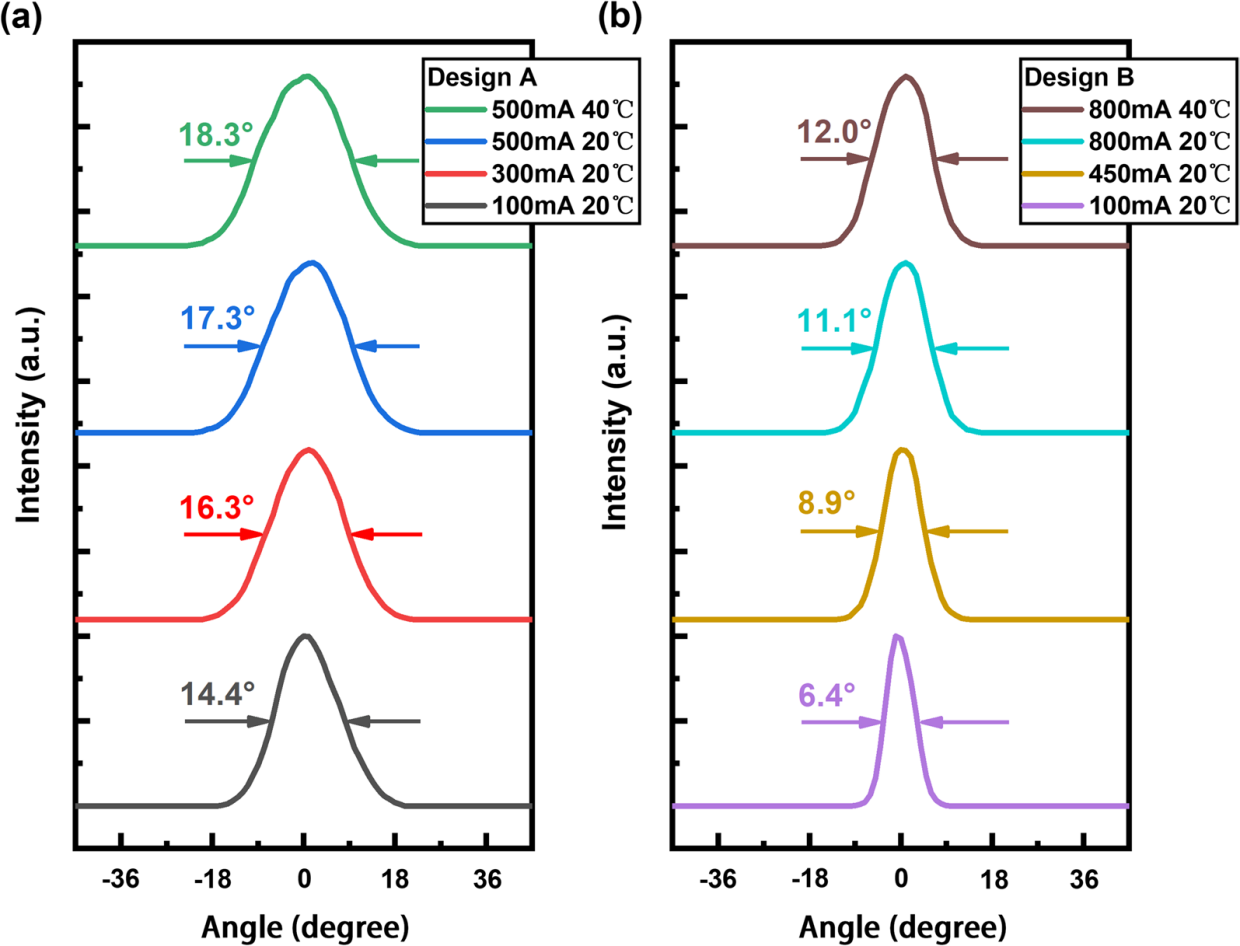
Lateral far-field intensity distributions of the RW laser designs at different injection currents and heat sink temperatures with corresponding FWHM beam angles indicated: **a** Design A and **b** Design B

The far-field characteristics measured using a rotating photoelectric detector for Designs A and B are presented in Fig. 5a and b, respectively, under different operating currents that were selected according to the P–I results presented in Fig. 4, where the corresponding FWHM lateral divergence angles are indicated. Single-lobe bell-shaped far-field distributions have been observed for both RW laser designs in the lateral and vertical directions over the entire ranges of operating currents considered, which demonstrated the high stability and purity of the fundamental-mode emissions of the RW laser designs. Large vertical beam divergences of 56$^\circ$ FWHM were obtained for both devices at the same 500 mA injection current regardless of the lateral waveguide structure, owing to the strong optical field confinement effect of the thin active layers of the GaSb-based laser epitaxial structure [1, 12]. For the lateral beam divergences, as demonstrated in Fig. 5, broadening occurred with Design A from 14.4$^\circ$ to 17.3$^\circ$ with an increasing injected current of 400 mA, while that of Sample B increased from 6.4$^\circ$ at 100 mA to 11.1$^\circ$ at 800 mA. This can be attributed to the heat-induced thermal lensing effect [26]. The results demonstrated a prominent reduction of Design B in lateral beam divergence compared to that of Design A at approximately identical current densities (B: 11.1$^\circ$ at 11.4 kA/cm$^2$ versus A: 17.3$^\circ$ at 12.5 kA/cm$^2$), indicating that the broadening near-field distribution of the lateral mode effectively translated into a narrower lateral far-field beam profile. Moreover, an increasing heat sink temperature from 20 to 40 $^\circ$C resulted in further broadened FWHM beam divergences of 18.3$^\circ$ and 12.0$^\circ$ of Designs A and B, respectively, attributed to the further exacerbation of the thermal waveguiding effect. The uniform Gaussian distributions sustained at 40 $^\circ$C proved the good thermal stability of the single-transverse-mode characteristics of both devices.

**Fig. 6:**
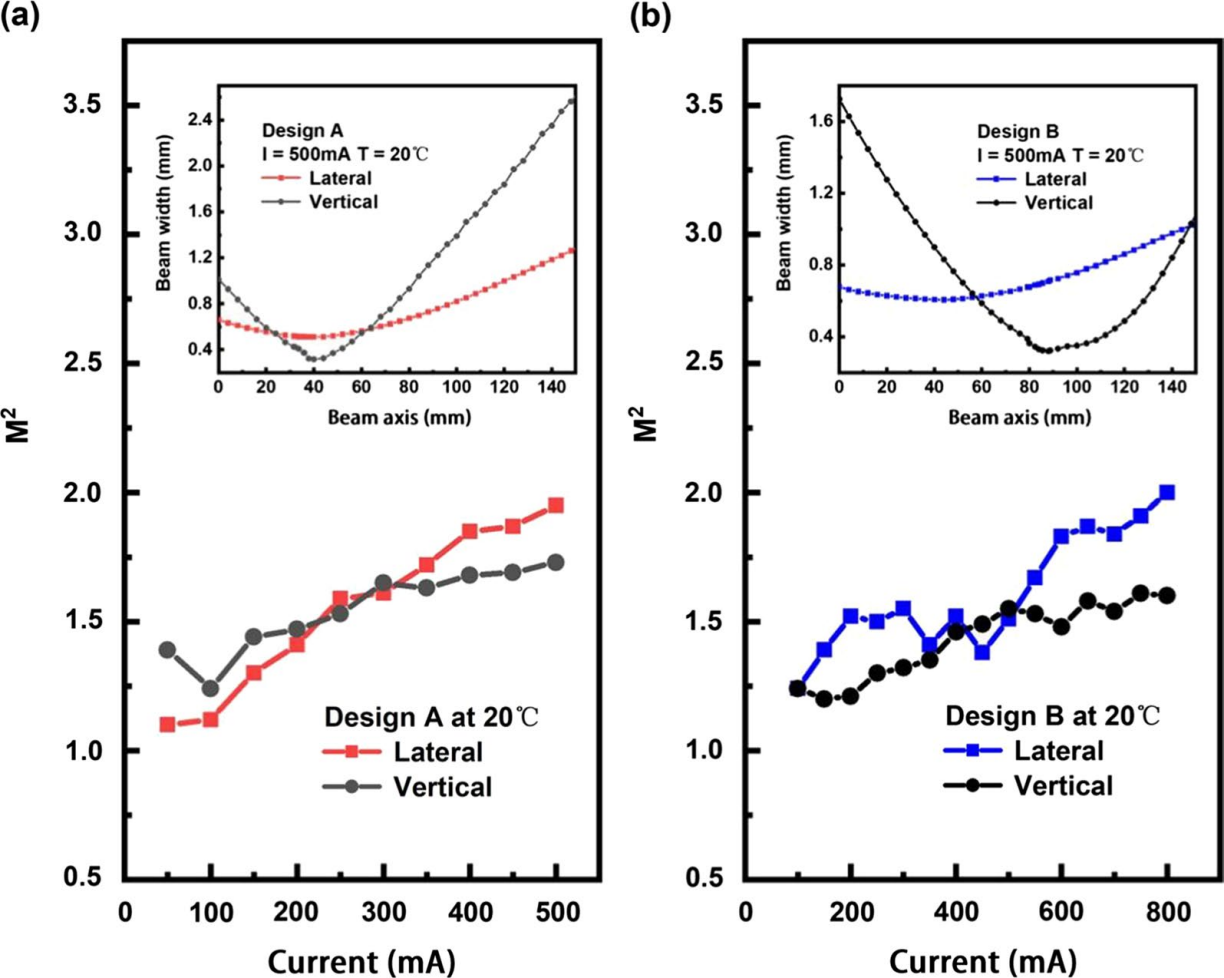
Measured beam quality factor M$^2$ values in the lateral and vertical directions versus injection current: **a** Design A and **b** Design B. The insets present the measured beam width in the lateral and vertical directions versus the beam axis obtained at a 500 mA injection current

The beam quality factor M$^2$ of Designs A and B were measured at different operating currents in the lateral and vertical directions using a commercial camera beam profiler that complied with the ISO 11146 recommended 4-standard-deviation (4-$\sigma$) method, where the laser beam was focused into an artificial beam waist using an aspherical collimating lens. The results obtained for Designs A and B are presented as a function of current in Fig. 6a and b, respectively. As indicated in the figures, the lateral and vertical M$^2$ values are less than 2 for both Designs A and B over the entire current range considered. Accordingly, both RW laser devices provided single-transverse-mode operation with high beam quality. Under these conditions, a maximum brightness of 21.5 MW $\times$ cm$^{-2}$ sr$^{-1}$ was obtained with Sample B at an 800 mA injection current. Meanwhile, Fig. 6 demonstrates that the M$^2$ values gradually increased with increasing injection current, demonstrating that the laser beams increasingly deviated from an ideal Gaussian shape [25]. These deviations can be attributed to modifications of the fundamental mode shape at higher currents and an increasing contribution of higher-order lateral modes. We further note that the variations of the lateral M$^2$ values with the current observed for Design A were much smaller than those observed for Design B. This demonstrated better discrimination against higher-order lateral modes attributed to the robust index-enhancement mechanism [23].

The insets of Fig. 6 present the beam widths measured in the lateral and vertical directions of the fabricated devices along the beam axes under injection currents of 500 mA. The beam waist locations of the lateral and vertical directions provide insight into the astigmatism characteristics of the different waveguide structures [13]. Accordingly, Design A provided considerably reduced astigmatism owing to the tighter lateral mode confinement. The low astigmatism characteristics of Design A imply that the vertical and lateral source points can be positioned simultaneously at the focal point of one single focus lens, which would facilitate the collimation and focus of this design for further utilization.

For comparison, we also fabricated an equivalently sized RW laser diode based on the conventional design with *w* = 5 $\upmu$m and *d* = 1.80 $\upmu$m, and subjected this device to the same performance testing applied to Designs A and B. The results obtained (not shown) demonstrated that the conventional RW laser diode design provided a single-transverse-mode output power exceeding 170 mW at a current of 600 mA with a far-field beam divergence of 16$^\circ$ and 56$^\circ$ FWHM in the lateral and vertical directions, respectively. In addition, M$^2$ values less than 2 were observed for the vertical and lateral directions over the entire current range considered, and noticeable astigmatism was observed. As would be expected, the conventional design offers reasonably good and balanced performance. However, the optimization routes adopted in the present study offer improvements in terms of specific optical characteristics that make the designs more suitable candidates for specific applications.

## Discussion

As demonstrated in the preceding section, both RW laser diode designs offer single-transverse-mode emission with excellent comprehensive performances. In addition to these, the nearly astigmatism-free characteristic and highly stable fundamental-mode operation of Design A make this design ideally suited for use in complicated optical systems that require beam transformation and propagation, while offering promising prospects as seed sources for various external cavity seeding applications. Moreover, the deeply etched RW employed in Design A makes this design compatible as well with other etched structures, such as curved waveguides or Bragg gratings, which facilitates the fabrication of SLD, DFB, DBR, and other devices. Meanwhile, the ultra-high single-transverse-mode power and brightness obtained by Design B offer promising prospects in pumping fiber amplifiers and solid-state laser systems. In addition, the reduced lateral beam divergence observed for Design B allows the lateral bulk-coupling or the use of relatively simple and low-cost optics for coupling into commercial single-mode fibers, such as SM1950 or PM1950 [10, 27], and photonic integrated circuit (PIC) applications [11].

More importantly, the selectively improved beam characteristics obtained via customized modifications in the waveguide structure according to the two proposed optimization routes provide substantial evidence supporting the validity and practicability of the waveguide design concepts of this work. Therefore, the waveguide parameter domain and the trends presented in Fig. 2 can be expected to further direct the design of GaSb-based RW laser devices to accommodate specific application demands with improved performance in the critical beam characteristics, such as high brightness, high optical power, low divergence, excellent beam quality, and low astigmatism. As inferences, RW laser diode designs with *w* < 4 $\upmu$m can be expected to provide an increasingly stable single-transverse-mode behavior with astigmatism-free characteristics, in compromise with the decreased optical power and broadened lateral far-field beam divergence. Moreover, the high lithography precision requirements would hinder the fabrication of the extremely narrow RW designs. Meanwhile, further enhancements in the brightness and optical power can be expected for designs of *w* > 7 $\upmu$m with further reduced values of *d*. However, the corresponding diminished effective index step may cause severe transverse mode instability owing to the predominant gain-guiding effect. Nonetheless, this problem may be solved by introducing structures that increase the threshold for higher-order modes and thereby stabilize the fundamental mode emission. Possible structures include leaky, corrugated, metallic, lateral absorbing, curved, flared, or highly resistive regions [21, 28, 29]. Relevant research will be conducted in the future.

## Conclusions

The present work has addressed the waveguide structure design factors that affect the single-transverse-mode beam characteristics of GaSb-based narrow RW lasers. The possible combinations of ridge widths and etch depths that support single-transverse-mode operation have been established, and two optimization routes of index-guiding enhancement and model gain expansion are indicated. These results were applied to develop two distinct optimized waveguide designs, denoted as Design A (*w* = 4 $\upmu$m, *d* = 2.05 $\upmu$m) and Design B (*w* = 7 $\upmu$m, *d* = 1.55 $\upmu$m). The impacts of the RW designs were experimentally evaluated in conjunction with 1-mm-long prototype laser diodes fabricated with optimized perpendicular ridge waveguides. Both RW laser diode designs were demonstrated to offer a high single-transverse-mode power > 150 mW with a high beam quality of M$^2$ < 2. In addition to these, the high stability and nearly astigmatism-free characteristic of Design A makes this design ideally suited for use in complicated optical systems that require beam transformation and propagation while offering promising prospects as seed sources for various external cavity seeding applications. Meanwhile, the extremely high single-transverse-mode power (258 mW), high brightness (21.5 MW $\times$ cm$^{-2}$ sr$^{-1}$), and narrow lateral divergence angle (11.1$^\circ$) of Design B offer promising prospects in pumping fiber amplifiers and solid-state lasers while facilitating the coupling into single-mode fibers or PIC applications.

## Data Availability

The datasets used and analyzed during the current study are available from the corresponding author on reasonable request.
